# Peptide receptor radionuclide therapy combinations for neuroendocrine tumours in ongoing clinical trials: status 2023

**DOI:** 10.7150/thno.91268

**Published:** 2024-01-01

**Authors:** Gianpaolo di Santo, Giulia Santo, Anna Sviridenko, Irene Virgolini

**Affiliations:** 1 Department of Nuclear Medicine, Medical University of Innsbruck, Innsbruck, Austria.; 2 Department of Experimental and Clinical Medicine, “Magna Graecia” University of Catanzaro, Catanzaro, Italy.

**Keywords:** PRRT, combination treatments, chemotherapy, neuroendocrine tumours, SSTR

## Abstract

A growing body of literature reports on the combined use of peptide receptor radionuclide therapy (PRRT) with other anti-tumuor therapies in order to anticipate synergistic effects with perhaps increased safety issues. Combination treatments to enhance PRRT outcome are based on improved tumour perfusion, upregulation of somatostatin receptors (SSTR), radiosensitization with DNA damaging agents or targeted therapies. Several Phase 1 or 2 trials are currently recruiting patients in combined regimens. The combination of PRRT with cytotoxic chemotherapy, capecitabine and temozolomide (CAPTEM), seems to become clinically useful especially in pancreatic neuroendocrine tumours (pNETs) with acceptable safety profile. Neoadjuvant PRRT prior to surgery, PRRT combinations of intravenous and intraarterial routes of application, combinations of PRRT with differently radiolabelled (alpha, beta, Auger) SSTR-targeting agonists and antagonists, inhibitors of immune checkpoints (ICIs), poly (ADP-ribose) polymerase-1 (PARP1i), tyrosine kinase (TKI), DNA-dependent protein kinase, ribonucleotide reductase or DNA methyltransferase (DMNT) are tested in currently ongoing clinical trials. The combination with [^131^I]I-MIBG in rare NETs (such as paraganglioma, pheochromocytoma) and new non-SSTR-targeting radioligands are used in the personalization process of treatment. The present review will provide an overview of the current status of ongoing PRRT combination treatments.

## Introduction

The current status of peptide receptor radionuclide therapy (PRRT) in neuroendocrine tumour (NET) patients involves a standardized treatment protocol with [^177^Lu]Lu-DOTATATE given in four cycles (7.4 GBq, eight weeks apart), under amino acid infusion for reduction of the absorbed kidney radiation dose 1,2. Increased availability through the industry based on FDA and EMA appropriation has led to an increased experience world-wide and to the incorporation of PRRT into various oncological guidelines 3-11. The clinical impact of somatostatin receptor (SSTR) positron emission tomography/computed tomography (PET/CT) 12 for the follow-up and clinical management was demonstrated for more than one third of NET patients 13. Furthermore, the role of [^18^F]F-FDG PET/CT in NET patients has been under debate in recent years, and several groups have evaluated the potential use of dual tracer PET/CT in NET patients 14-16 resulting also in prognostic grading proposals 17.

The first disease control data with [^177^Lu]Lu-DOTATATE in gastroenteropancreatic (GEP)-NET patients were already published in 2008 by the Rotterdam group 18 who also reported similar effective long-term data for bronchial carcinoids in 2017 19 with a median overall survival (OS) ranging from 52 to 71 months. In the Phase 3 NETTER-1 study 2, with a median follow-up of >6.3 years, the pre-specified final analysis of OS in the intention-to-treat population did not reach statistical significance (HR, 0.84 [95% CI: 0.60, 1.17]; p = 0.30, two-sided) which was potentially impacted by a high rate of cross-over (36%) of patients in the control arm to PRRT. The median OS was 48.0 months in the [^177^Lu]Lu-DOTATATE arm and 36.3 months in the control arm (i.e. 11.3 months survival benefit). The NETTER-1 safety data showed a low incidence of long-term side-effects regarding haematotoxicity and nephrotoxicity (6/111 (5.4%) patients had ≥Grade 3 nephrotoxicity, no new cases of myelodysplastic syndrome (MDS) or acute myeloid leukemia (AML)). In our own retrospective long-term study report 20 the median OS was 9 years for responders and 2 years for non-responders, and one third of patients was still alive after the ≥12-year follow-up.

The European Association of Nuclear Medicine (EANM) focus guideline 11 suggests PRRT as a) first line treatment in non-resectable or disseminated NET in a minority of highly selected patients with high SSTR-expression (based on risk and symptoms, primary tumour location), b) PRRT as second line treatment for GEP-NET, if there is sufficient SSTR-expression in all lesions, c) consideration of PRRT in GEP-NET G1 and G2 (Ki-67 <20%) patients at first disease progression when all lesions are matched in [^68^Ga]Ga-DOTA-SSTR / [^18^F]F-FDG PET/CT and d) PRRT in a minority of patients with G3 NET (Ki-67 >20%), preferably within clinical study protocols, such as in combination with capecitabine (CAP) and temozolomide (TEM). Re-challenge PRRT should be considered in patients with disease stabilization or remission for at least one year after end of first PRRT. Our own 14, 21 and other 22, 23 data strongly suggest that re-challenge PRRT can be as effective as the first course of PRRT with similar safety profile.

The combined use of PRRT with other anti-tumour therapies anticipates synergistic effects with perhaps increased safety issues. Only a few studies have so far evaluated the combination of PRRT with other treatments such as cytotoxic chemotherapy or molecular targeted treatments. Generally, combination treatments to enhance PRRT outcome are based on either improved tumour perfusion, upregulation of SSTR or radiosensitization with DNA damaging agents or targeted therapies 24.

Several Phase 1 or 2 trials are currently recruiting patients in combined regimens. Here we overview the current status of ongoing PRRT combination therapies.

### Role of diagnostic imaging in combination treatments

NETs are heterogeneous neoplasms exhibiting intra- and inter-lesion variability that could impact treatment success and prognosis. The growing knowledge about different imaging pathways as well as the progressive introduction of new radiopharmaceuticals for diagnosis and treatment could provide a real whole-body “*in vivo*” study to characterize each lesion and its receptor expression 25. In other words, molecular images could overcome the limitation of a single-site biopsy, giving a better understanding of disease variability, and guiding to the best treatment option for each patient 26. In clinical practice, advances in molecular imaging using dual tracer PET with [^68^Ga]Ga-DOTA-SSTR and [^18^F]F-FDG are emerging as a potential tool to investigate lesion differentiation, affecting patient management 27. Namely, highly SSTR-avid (WHO G1/G2) NETs are usually treated with octreotide long-acting repeatable (LAR) followed by PRRT, whereas highly FDG-avid (WHO G3) NETs are commonly treated with chemotherapy. However, an intermediate “gray zone” exists, represented by those patients with high uptake on both [^68^Ga]Ga-DOTA-SSTR and [^18^F]F-FDG dual tracer PET/CTs 28. For these patients, a therapeutic approach combining PRRT with chemotherapy may represent an effective strategy. As shown in Table 1A and B, most of the published data about the combination of PRRT plus chemotherapy include in the baseline assessment [^18^F]F-FDG PET/CT beyond SSTR expression. For instance, Nicolini et al. in their prospective study included [^18^F]F-FDG positive patients with a SUV >2.5 in at least one documented lesion 29. Also, the Australian group used dual tracer imaging as part of the baseline assessment 30,31 showing a 27% of complete metabolic responders on [^18^F]F-FDG PET/CT after combined treatment. Similar results were also shown by Yordanova et al. 32. This deserves a mention in order to underline the possible impact of combination therapies in [^18^F]F-FDG positive patients, who did not respond to PRRT alone, and the need for comparative studies for this setting of patients.

Moreover, the thera(g)nostics concept is not limited to the use of [^18^F]F-FDG, but could also be extended to the other combined treatments, running from the “old” use of [^123/131^I]I-MIBG to other potential targets including the gastrin releasing peptide receptor (GRP-R) 33, or glucagon-like peptide 1 (GLP-1) 34, as well as the fibroblast activating protein (FAP) 35. In addition, the immuno-PET could pave the way for the study of PD-1/PD-L1 expression 36 and the poly (ADP-ribose) polymerase-1 (PARP1) imaging-based analysis was also proposed 37. These possibilities could change the perspective of NET patients, allowing better patient selection, response prediction and follow-up, adapting treatment to the characteristics of each patient during the course of the disease.

## Combination of PRRT with Chemotherapy (Figure 1; Table 1A, B)

The role of chemotherapy in NETs has evolved in recent years. Radiosensitizing low-dose chemotherapy may exert its effect via inhibition of DNA repair, cell proliferation arrest, increased DNA damage, or apoptosis. The CAPTEM regimen demonstrated significant anti-tumour activity in both pancreatic (p) NETs 38 and non-pancreatic NETs following several retrospective studies 39-42. Owen et al. 41 demonstrated for pNET as well as non pNET a progression free survival (PFS) of 13 months and an OS of 29.3 months. In this study, partial remission (PR) was seen in 11 (38%), stable disease (SD) in 15 (52%) of patients giving a disease control rate (DCR) of 90%. A trend of increased response rate in patients with low O^6-^methylguanine DNA methyltransferase (MGMT) activity was also seen. On the other hand, in the study of Cives et al. 43 response to CAPTEM was not influenced by MGMT, proliferative activity or alternative lengthening telomeres (ALT) pathway activation.

A retrospective critical analysis of the cytoreductive impact of systemic therapies in advanced pNETs identified a multi-agent chemotherapy in combination with PRRT as probably the best treatment strategy 44. In an initial study on the combination of [^177^Lu]Lu-DOTATATE-PRRT with CAP in 7 patients, no Grade 4 haematotoxicity was reported and Grade 3 thrombocytopenia in one single patient only 45. One of the first retrospective reports by the Melbourne group after high activity [^111^In]In-pentetreotide PRRT concluded that re-challenge PRRT with [^177^Lu]Lu-DOTATATE with either radiosensitizing infusional 5-Fluorouracil (5-FU, n=27) or CAP (n=2) is safe and well-tolerated with an OS of 34 months 46. However, caution was recommended in patients with bone metastases with one single case of Grade 4 lymphocytopenia only. Hence, the authors concluded that in terms of safety, also the sequence of [^177^Lu]Lu-DOTATATE plus radiosensitizing chemotherapy after [^90^Y]Y-DOTATOC-PRRT should be safe 47 as Grade 3 or 4 haematotoxicity was found only in 3.6% in a large number of patients after re-challenge PRRT with [^177^Lu]Lu-DOTATATE 48. Renal failure as the dose-limiting factor did not appear, and this probably is somewhat higher in a combination with [^90^Y]Y-based-PRRT 49.

A Phase 2 study 50 in 33 metastatic NET (mNET) patients treated with PRRT plus CAP (1650 mg/m^2^/d/14d) revealed 24% PR, 70% SD (i.e. DCR 94%) despite one single case of Grade 3 thrombocytopenia and 3 cases of Grade 3 angina. The same group demonstrated for on top-treatment with escalating doses of TEM (100-200 mg/m^2^/5d) no dose-limiting toxicities 51. The commonest toxicities were Grade 3 nausea in 1 (3%) patient, Grade 3 neutropenia in 2 (6%) patients and Grade 3 angina in other 2 (6%) cases. Complete remission (CR) was achieved in 15%, PR in 38%, SD in 38% (DCR 91%). PFS was 31 months, OS was not reached at the 24-month follow-up. In 30 patients with pNET the same authors demonstrated an overall response rate of 80% (CR in 13%, PR in 67%), PFS 48 months, OS was not reached at the 33-month follow-up. In this study Grade 3 thrombocytopenia was observed in 10% of patients 52.

Nicolini et al. 29 reported for the combined use of PRRT (5x5.5 GBq) and CAP (1000 or 1500 mg/d) in 37 patients, assessed by dual-tracer PET/CT (Ki-67≤ 55%), haematotoxicity Grade 3 or 4 in 16.2% of patients, diarrhea in 5.4% and asthenia/fatigue in 5.4%. PR was seen in 10 patients (30%), SD in 18 patients (55%) and DCR in 85%, PFS was 31.4 months and OS was not reached at the 38th month of follow-up.

Kong et al. 30 assessed predictors of response and long-term survival following radiosensitizing infusional chemotherapy with 5-FU (200 mg/m2/24h) in combination with PRRT. A high proportion (70%) of patients received benefit from the treatment with OS rates at 2 and 5 years of 72.1% and 52.1%, respectively. Patients with pNET and lesions >5 cm appeared to have a lower objective response rate, thus needing a more aggressive approach. The same group previously reported under the same treatment regimen a complete metabolic response in 27% of FDG-avid patients and a biochemical response (i.e.>25% chromogranin-A decrease) in 45% of patients 31 and for patients with Ki >55% a PFS of 4 months and OS of 7 months 53.

Chauhan et al. 54 reported in 12 patients with unknown origin a PFS of 10.8 months for Grade 2 and 7 months for Grade 3 mNET patients. In this study, one patient developed the foot-hand syndrome Grade 3.

Yordanova et al. 32 reported for 15 patients with mNET a PFS of 7.1 months and OS of 25.3 months. The DCR was 55% when assessed by CT, 28% by [^18^F]F-FDG PET/CT and 44% by [^68^Ga]Ga-DOTATOC PET/CT.

The Australian study 55, 56 reported for pNETs a PFS of 61.1% when treated with PRRT+CAPTEM versus 33.3% for patients treated by CAPTEM only at the 27-month follow-up. In their evaluation, no difference was found for mNETs at the 36-month follow-up between both groups. However, the long-term results are not yet published.

Several other studies are currently recruiting NET patients combining PRRT with chemotherapy, particularly with CAPTEM 57-61

New radioisotopes such as [^225^Ac]Ac-DOTATATE in combination with CAP (2 g/d, days 1-14) may impact future PRRT outcomes 62. In this study the authors reported an improved OS also for patients who received prior [^117^Lu]Lu-DOTATATE. A poorer OS was estimated for patients with bone metastases by multivariate analysis while the OS probability at the 24-month follow-up was 70.8%.

### Conclusion on PRRT plus Chemotherapy

Although prospective data are needed, PRRT in combination with chemotherapy seems to become an effective treatment option in patients with a wide variety of advanced metastatic NETs. The indication for combined treatment may be based on FDG-positivity and/or Ki-67 values greater than 20%, basically Grade 3 disease. No clinically significant toxicity has been reported so far for this combination, even in patients who have failed prior conventional therapies, except of one case of neutropenic sepsis 63. The DCR seems to come up >90% depending on the response criteria used.

## Combination of PRRT with PARP Inhibitors (Table 2)

Rendering the tumour cell more sensitive to radiation is one treatment strategy. Thus, inhibition of the DNA repair mechanism that repairs the DNA damage induced by radiation of PRRT could be a new treatment basis. The DNA damage consists of double- and single-strand DNA damages that are repaired by poly (ADP-ribose) polymerase-1 (PARP1) 64. Hence, inhibitors of PARP1 (PARP1i) have become an important tool for inhibition therapy especially in BRCA1/2^mut^ patients, leading to cytotoxicity which is termed “*synthetic lethality*”. In this context, the targeting of PARP1 has emerged as a nuclear imaging and thera(g)nostic modality. PARP-imaging agents may be potentially useful as a guidance to predict therapy response to PARP1i, or to monitor therapy response. In the past, several radiolabelled compounds were synthesized and also used in preclinical and clinical studies 65. Among the tracers reported ^18^F-olaparib 66, a direct analog of olaparib, has recently gained significant attention and the alpha emitting compound [^211^At]At-MM4 67 has shown encouraging anti-tumour activity despite of methodological challenges regarding radiochemistry on the one, and biodistribution, on the other hand.

Recently, PARP1i in combination with [^117^Lu]Lu-DOTATATE increased the anti-tumour activity in experimental animals 68, 68 as well as in human NET cells 70, 71.

Three Phase 1/2 studies are currently recruiting using escalating doses of either olaparib 72-74 or talazoparib 75 in combination with PRRT.

### Conclusion on PRRT plus PARPi

Based on the preclinical studies the combination with PARPis has immense clinical potential and results of the ongoing clinical trials remain to be awaited.

## Combination of PRRT with Immune-Checkpoint-Inhibitors (ICIs) (Table 3)

ICIs are considered to be a revolutionary treatment option in the field of primarily solid tumours and are increasingly used in multiple other tumour types 76, 77. As for NET, only limited data are available. In an animal model Esfahani et al. 78 recently demonstrated that the combination of PRRT and anti-PD1 treatment with pembrolizumab showed the most robust inflammatory response to NETs and a better overall outcome than ICIs or PRRT alone. The most effective regimen is PRRT preceding anti-PD1 administration by several days. The success of a combination treatment of PRRT with ICIs may be ruled out by prior immuno-PET, a new methodology emerged as a promising imaging tool for the prediction of response as well as the monitoring of response to treatment 79. As both, ICIs and PRRT are registered products today, this approach is now translating into the clinic.

No dose limiting toxicity was observed in an initial small cohort of 9 NET patients reported by Kim at al. 80 with predominantly small cell lung cancer. The patients were treated by PRRT at half dose (3.7 GBq) in combination with nivolumab (days 1 and 15; 240 mg i.v.). In this heavily pretreated cohort one patient showed PR and two patients SD. This study is still recruiting 81.

Other studies are on the way including PRRT in combination with avelumab 82 or pembrolizumab 83 in Merkel Cell Cancer patients. A study combining PRRT and pembrolizumab versus pembrolizumab plus transarterial embolization (TAE) or pembrolizumab plus radioembolization (RE) for liver metastatic NET with a Ki-67 >20% is also active 84.

### Conclusion on PRRT plus ICIs

Based on the preclinical studies the combination with ICIs has immense clinical potential and results of the ongoing clinical trials remain to be awaited.

## Combination of PRRT with Inhibitors of Ribonucleotide Reductase, DNA-dependent Protein Kinase, Tyrosin Kinase (TKI) or DNA Methyltransferase (DNMT) (Table 4)

The ribonucleotide reductase inhibitor triapine 85 is currently tested in a PRRT combination study in mNET 86, 87. The DNA-dependent protein kinase inhibitor peposertib 88 is tested in combination with PRRT in pNET 89. The tyrosin kinase inhibitors sunitinib 90 and cabozantinib 91 are also tested in combination with PRRT. On the basis of SSTR2 upregulation also DNMT inhibitors such ASTX727 (i.e. cedazuridine and decitabine) is currently tested in combination with PRRT 92.

### Conclusion on PRRT plus Anticancer Drugs

Based on the preclinical studies the combination with anticancer drugs has immense clinical potential and results of the ongoing clinical trials remain to be awaited.

## Improvement of PRRT by Different Concepts (Table 5)

### Combination of PRRT with [^131^I]I-MIBG

[^131^I] I-MIBG, a guanethidine analog of norepinephrine, has been used for the treatment of paraganglioma, pheochromocytoma and neuroblastoma as well as other NETs over decades. The “old” strategy of [^123/131^I]I-MIBG 93-95 is currently challenged by the new treatment paradigm of [^68^Ga]Ga / [^177^Lu]Lu- or [^90^Y]Y-SSTR-based thera(g)nostics 96,97, or supposedly also a combination of both. However, results are difficult to interpret as reports are mostly retrospective with low patient numbers only 11. A recent Phase 1 study in a limited number of patients investigated the combined use of [^131^I]I-MIBG with [^90^Y]Y-DOTATOC in a dose escalating manner 98. The calculated absorbed tumour dose estimates suggested an increase of 34 to 83% for the combination as opposed to PRRT alone with dose limits of 19 Gy to the kidneys and 0.15 Gy to the bone marrow. No dose-limiting toxicities were observed despite of one case Grade 3 thrombocytopenia.

**Figure 2:**
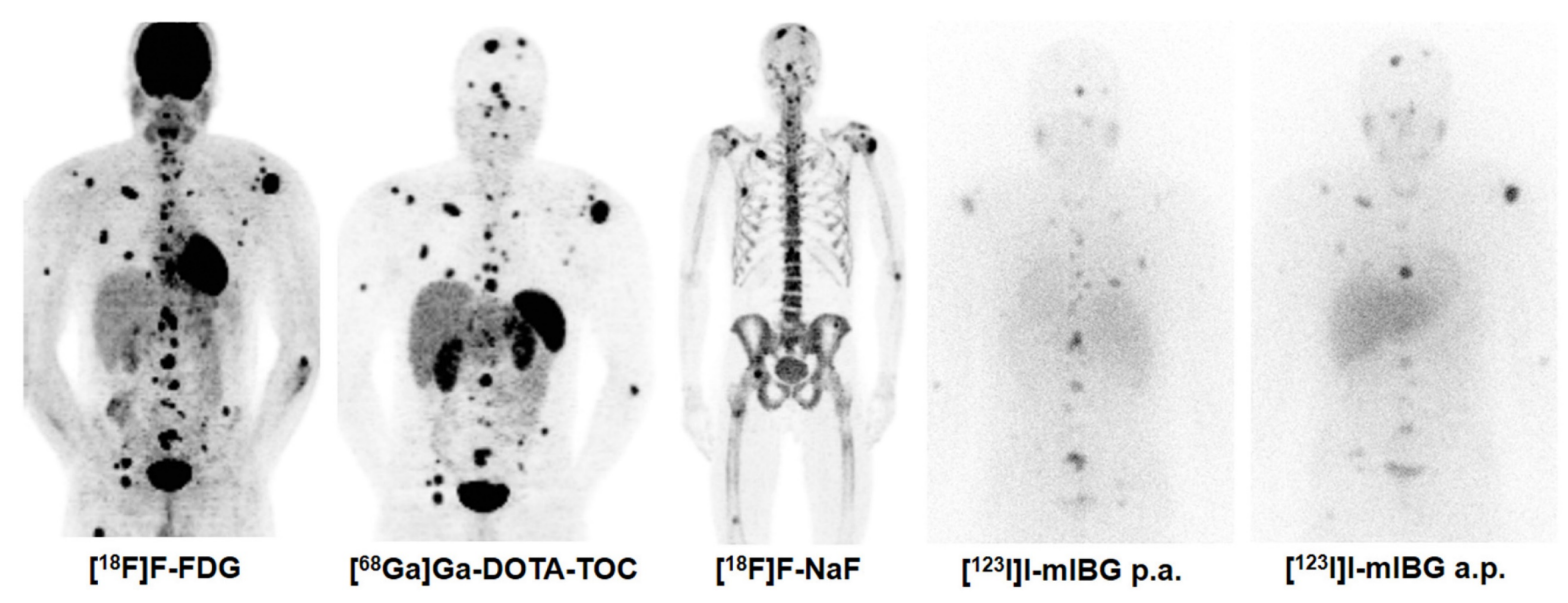
**Combination Treatment of PRRT with [^131^I]I-mIBG**. [^68^Ga]Ga-DOTA-TOC, [^18^F]F-FDG, [^131^I]I-mIBG, and [^18^F]F-NaF imaging in a 30 year old male patient with metastatic pheochromocytoma (functional tumour). In such rare cases, the combination treatment of PRRT with [^131^I]I-mIBG can be considered based on tumour accumulation of both thera(g)nostics.

A clinical trial evaluating the safety of PRRT in combination in with Azedra(R) to treat mNET (SPORE-3) is currently active 99. No other clinical trials are currently on the way and such a combination remains for individual cases based on positive imaging results as presented in **Figure 2.**

### Combination of PRRT with Neoadjuvant Surgery

Early PRRT can be applied for downstaging of the disease or as neoadjuvant treatment in order to make resection possible or improve the cure rate. Mainly reported for pNETs, neoadjuvant PRRT may also result in fewer surgical complications 100, 101.

Recently, Minczeles et al. 102 reported that early administration of PRRT followed by surgery is associated with favourable long-term outcomes in patients with locally advanced or oligometastatic pNET and can be considered for selected patients with vascular involvement and/or increased risk of recurrence. Two clinical studies are currently recruiting patients with either GEP-NET 103 or pNET 104 receiving neoadjuvant PRRT followed by surgery.

**Figure 3:**
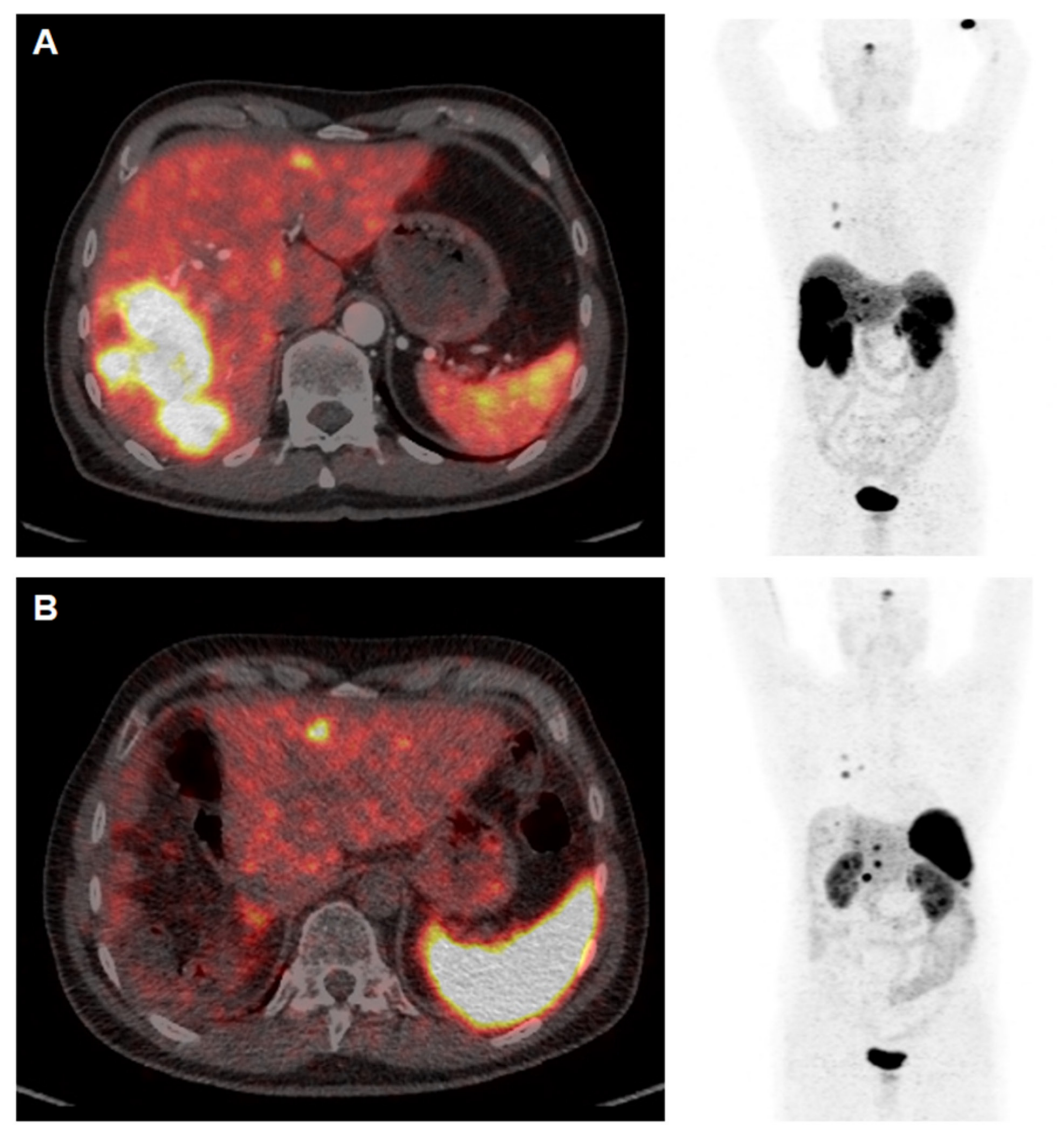
**[^68^Ga]Ga-DOTATOC PET/CT Study Prior to (A) and 12 weeks after (B) Cytoreductive Surgery**. The patient (64 y) was diagnosed with medullary thyroid cancer (MTC) at stage pT1a N1a R0 Mx in 2005. After complete thyroidectomy in July 2005 the disease was stable until 2014 when SSTR-positive liver metastases were diagnosed. PRRT with [^177^Lu]Lu-DOTATATE (accumulated activity 29.47 GBq) then resulted in disease stabilisation until January 2022. Appearance of increased size of liver metastases (A) were associated with increased episodes of watery diarrhea, not responsive to symptomatic therapy. In April 2023 the patient underwent cytoreductive surgery (B) and currently receives a postsurgery second period of [^177^Lu]Lu-DOTATATE PRRT. Symptoms are completely relieved and the patient has gained >10 kg body weight (September 2023).

We include a clinical case of a patient with medullary thyroid cancer with mainly liver metastases who improved dramatically with the combination of cytoreductive surgery and PRRT (Figure 3).

### Increasing PRRT Results by Addition of Long-Acting SST Analogs

The anti-proliferative effect of somatostatin (SST) analogs was established in the PROMID 105 and CLARINET 106 trials, and today PRRT is usually applied in combination with long-acting SST analogs. However, this basic combination therapy is not well established. Yordanova et al. 107 reported higher tumour response rates, especially in patients with higher tumour burden or higher Ki-67 values for the combination group. The results are controversial and no final statement was ever discussed 108-110.

### Increasing PRRT Results by (Individualized) Dosing - Dosimetry

Basically, today dosimetry is not (anymore) a prerequisite for PRRT as we have come from single photon emission tomography (SPECT)/CT to PET/CT. However, for small-sized lesions (especially <2 cm) SSTR PET/CT increases the so-called “Krenning Scores” which were historically introduced to define patients for treatment with PRRT using [^111^In]In-DTPA-D-Phe1-octreotide 111. Furthermore, Roth et al. 112 reported that the tumour absorbed dose decreases from cycle 1 to cycle 2 of PRRT by 6% for G1 tumours and 14% for G2 tumours. This may be caused by lower uptake in the tumour lesions and decreasing tumour volume as response to treatment. There are also estimations that a cumulative dose <29.6 GBq of [^177^Lu]Lu-DOTA-TATE is less efficacious in terms of tumour response and survival compared to patients receiving 29.6 GBq 113. Dosimetry-based personalization of PRRT by increasing the injected activity until an absorbed kidney dose of 23 Gy has shown that a high proportion of patients is probably undertreated 114 using the one-size-fits-all regime 1, 2.

The debate on dosimetry cannot easily be solved 115. While for the clinical setting, the “real world” use of PRRT, dosimetry may play a minor role for most patients, accurate dosimetry seems important in patients receiving PPRT combinations.

### Increasing PRRT Results with New SSTR-Based Peptides/Radioligands

New SSTR-based radioligands include the group of antagonists. Among these, [^177^Lu]Lu-OPS201 116, 117 and LM3 118 have shown increased in vitro and in vivo binding to NET tumours despite of decreased kidney absorbed dose. By introducing an albumin binding moiety, the bioconjugate-modified [^177^Lu]Lu-EB-TATE has recently shown clinical potential 119, 120 in terms of increased SSTR-based tumour uptake. The concept of [^64/67^Cu]Cu-thera(g)nostics has so far had no real breakthrough due to technical production problems 121.

### Increasing PRRT Results with New Isotopes, i.e. Alpha

Alpha-labelled SSTR-based analogs have recently gained increased interest in the clinical setting. The [^161^Tb]Tb-labelled SSTR antagonist LM3 currently is investigated in a combination setting with [^177^Lu]Lu-DOTATOC 122. The new SSTR ligand [^211^Pb]Pb-DOTAMATE has also gained attraction in an ongoing clinical study 123. Other possibilities in NET include [^225^Ac]Ac-labelled compounds such as [^225^Ac]Ac-DOTATATE 124 which has already shown good tolerability in PRRT-naïve patients 125. “Dual PRRT” in general is based on combining SSTA analogs radiolabelled with different isotopes. Such combinations were [^90^Y]Y-labelled with [^177^Lu]Lu-labelled analogs in the past 126, 127 and nowadays a move towards a combination with alpha-emitting isotopes is registered based on higher energy transfer and lesser penetration range. In these combinations the lower energy and shorter tissue penetration range of maximal 2-4 mm for [^177^Lu]Lu products and of maximal 11 mm for [^90^Y]Y products are combined for large and bulky NET metastases. While several results for this combination are promising from the past 126, 127 the study NCT04029428 128 is currently recruiting 150 NET patients of various origin using [^90^Y]Y (4x 3.7 GBq, [^177^Lu]Lu (4x5.55 GBq) or the mixed PRRT combination.

### Increasing PRRT Results by Combination of Intravenous and Intraarterial Routes of Administration

Several attempts have been made in liver predominant disease to use the liver-directed intraarterial route for PRRT application in combination with the intravenous route of PRRT application 129. As about two thirds of NET patients have metastatic liver disease and as the tissue penetration of [^177^Lu]Lu-DOTATATE is only 2-4 mm radioembolization of especially larger liver metastases with [^166^Ho]Ho or [^90^Y]Y seems to be meaningful. As the technique may lead to severe hepatotoxicity along with radioembolization, a multidisciplinary team is essential in the decision making process. Kratochwil et al. 130 reported successful results after intraarterial administration of [^213^Bi]Bi-DOTATOC in patients with liver metastases resistant to [^90^Y]Y/[^177^Lu]Lu-DOTATOC. Two studies are currently evaluating the combination of i.v. and i.a. routes of PRRT 131,132.

### Increasing PRRT Results by Use of New Non-SSTR-based Radioligands

**Figure 4:**
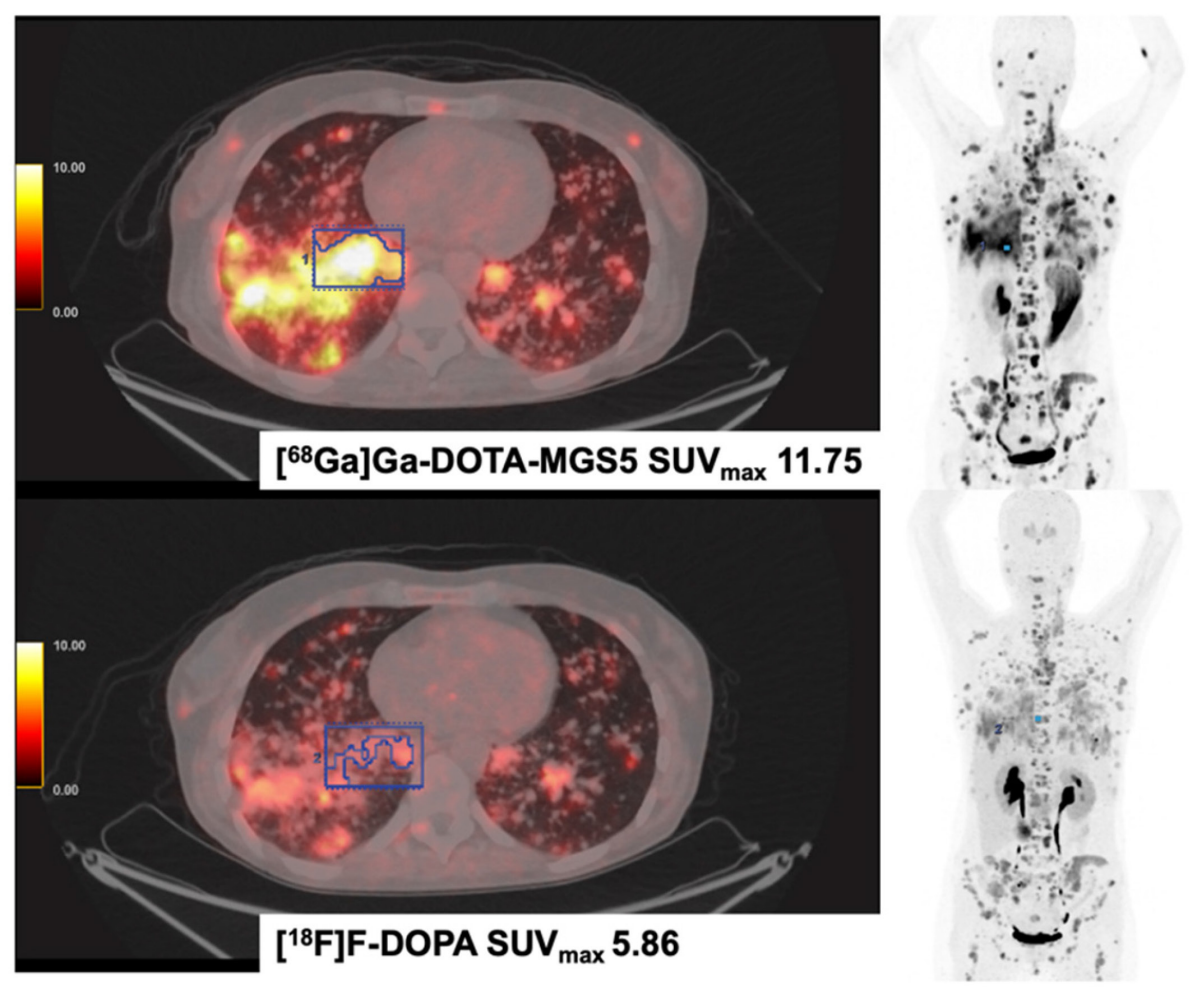
** [^68^Ga]Ga-DOTA-MGS5 (DOTA-DGlu-Ala-Tyr-Gly-Trp-(N-Me)Nle-Asp-1-NaI-NH_2_) and [^18^F]F-DOPA PET/CT in a Patient with Medullary Thyroid Cancer (MTC).** The patient was diagnosed with medullary thyroid cancer in 2014 (Ki-67 15%). Disease progression was evidenced after treatment with sorafenib, vandetanib, cabozantinib and long-acting octreotide. In 2023, disseminated metastases were seen in both examinations, [^18^F]F-DOPA and [^68^Ga]Ga-MGS5 PET/CT. The patient currently receives treatment with the CCK2-targeting [^177^Lu]Lu-labelled antagonist [^177^Lu]-Lu-PP-F11N (DOTA-(DGlu)6-Ala-Tyr-Gly-Trp-Nle-Asp-Phe-NH2) in Basel, Switzerland.

Several non-SSTR-receptors have been addressed to be potential targets for PRRT 133. Such potential targets include the gastrin releasing peptide receptor (GRP-R), cholecystokinin receptors (CCK2) or glucagon-like peptide 1 (GLP-1). The NEORAY Phase 1 study is currently recruiting patients with solid tumours 134. The CCK2R antagonist [^177^Lu]Lu-PP-F11N has recently shown clinical safety in patients with medullary thyroid cancer (MTC) 135, and further studies are under way including our own derivative [136**; Figure 4**]. Other new peptide tracers are promising such as exendin in insulinoma 137, and novel therapy concepts are to be expected.

Furthermore, the present “hype” of thera(g)nostics for fibroblast activating protein (FAP) 138 may be adopted also for NET patients not responsive to PRRT with SSTR compounds.

## Conclusions on the Improvement of PRRT by Different Concepts

The published data are mostly retrospective, limited patient numbers, and uncontrolled. Data on the use of long-acting octreotide or lanreotide from Phase 3 studies have led to their incorporation in the standard setting of PRRT combinations. Dosimetry and/or individualized dosing does not hold in the so-called “real life experience”. New antagonists, especially when labelled with alpha-emitting radioisotopes, seem most promising in the future. For further personalization of PRRT, the combination of intravenous and intraarterial routes of application seem reasonable in individual cases as does the use of new radioligands.

## Perspectives

Three multicenter Phase 3 trials are currently active or still recruiting 139-141. The COMPETE study 141 evaluates the efficacy and safety of [^177^Lu]Lu-edotreotide PRRT (4 cycles, 12 weeks apart) in Grade 1 and 2 GEP-NET patients versus everolimus (10 mg/d) whereas the COMPOSE study 139 evaluates against best standard of care in Grade 2 and 3 GEP-NET patients. Efficacy and safety results in Grade 2 and Grade 3 GEP-NET patients under PRRT with [^177^Lu]Lu-PRRT (4 cycles, 8 weeks apart) in combination with long-acting octreotide versus high dose octreotide are evaluated in the NETTER-2 trial 140. The results of these three larger studies may lead to a broader application of PRRT in the near future, especially in G3 NET patients. As experimental preclinical studies with PARPis and ICIs are very promising, the results of the ongoing clinical studies in patients are to be anticipated. Furthermore, the combination results of sunitinib as a potential radiosensitizer for PRRT patients are to be awaited soon. The future is bright with all other combinations in prospective studies using various other modern anti-tumour substances. Certainly, further personalization of PRRT combinations on the one hand, and clinical safety of these combinations at the same time on the other hand, remain the major challenge. The treatment of NET patients is complex due to the heterogeneity of the disease and the current different combination possibilities tested in a variety of clinical studies are highly valuable. In this scenario, the possibility of the translating thera(g)nostic concept to clinical reality also using non-SSTR-receptors as well as immuno-PET can pave the way for the application of new combined treatments strategies. This gains increased importance considering the chance to choose a “tailored” treatment option for every NET patient in the era of personalized medicine. Last but not least, the ongoing development of combined treatments underlines the need for a multidisciplinary approach, not only in terms of treatment strategies, but also in terms of skills and knowledge. Therefore, it is crucial to train specialists who take care of the patient in every medical aspect, especially when using combined treatments.

## Consent to publish

Informed consent for the publication of images was received from all participants who appear in the manuscript.

## Figures and Tables

**Figure 1:**
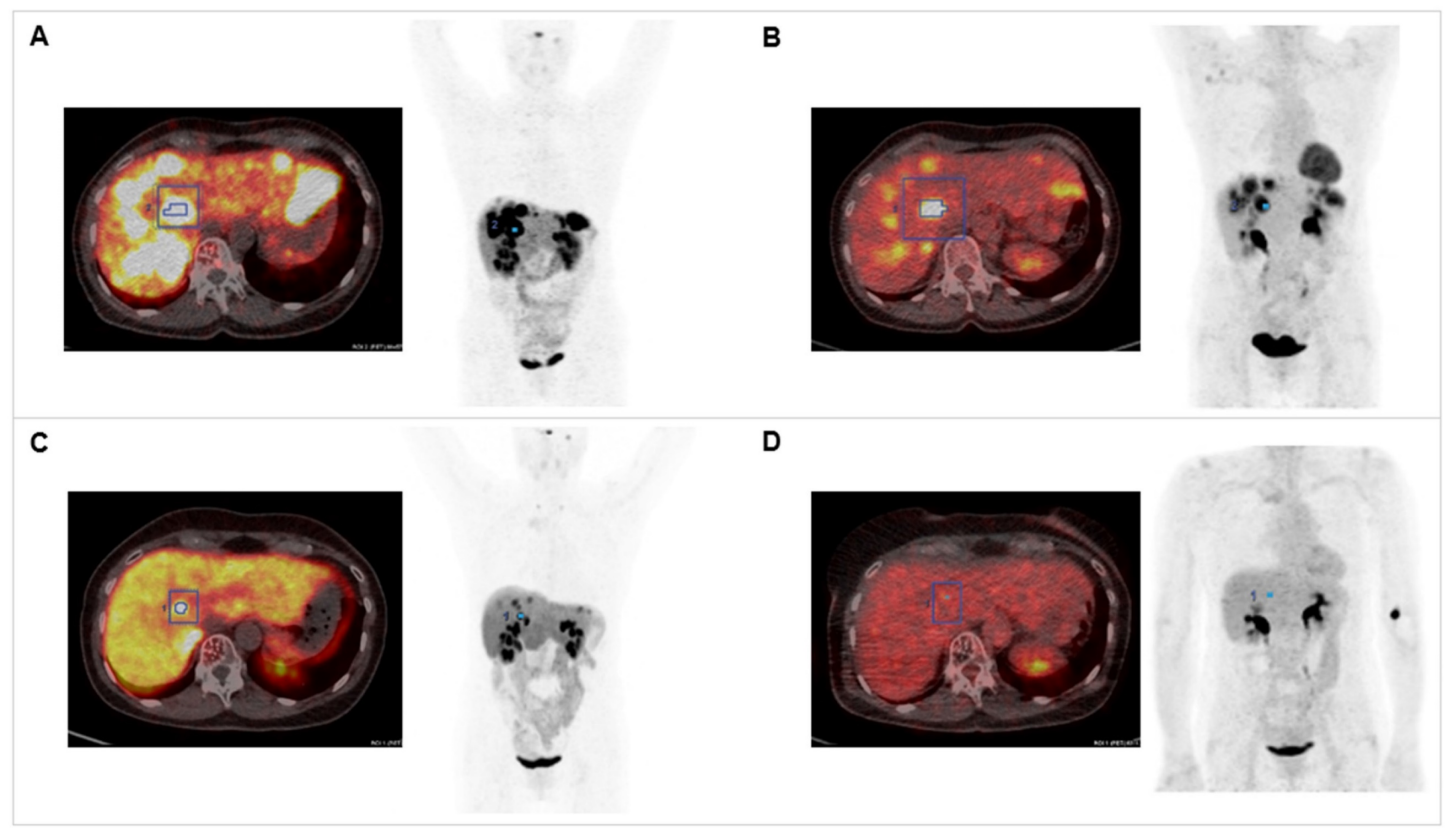
** Combination of PRRT with CAPTEM.** The patient was diagnosed with pancreatic NET (Ki-67 15%, pT3N0M0R0) following partial pancreatectomy in 2012. Dual tracer PET/CT with (A) [^68^Ga]Ga-DOTATOC and (B) [^18^F]F-FDG indicated multiple liver metastases in 2020. The combination treatment with 1500 mg/m^2^ capecitabine (CAP) and 200 mg/m^2^ temozolomide (TEM) with ^177^Lu[Lu]Lu-DOTATATE (accumulated activity 29.54 GBq) resulted in partial response in (C) [^68^Ga]Ga-DOTATOC PET/CT (SUV_max_ decreased from 57.43 to 24.92) and (D) complete response in ^18^[F]F-FDG PET/CT (SUV_max_ decreased from 14.51 to 4.76).

**Table 1A:** Combination of PRRT with CHEMOTHERAPY

Treatment Combination	n	Patients	Safety	Results	Reference
**PRRT (5x5.5GBq) + CAP (1000 or 1500 mg/d/14d)** [^111^In]In-Pentetreotide [^68^Ga]Ga-DOTATOC PET/CT [^18^F]F-FDG PET/CT	37	GEP-NET (1-3, Ki<55%)	4xG3/4 neutropenia 1xG4 thrombocytopenia 1xG3 lymphopenia fatigue 5.4% diarrhea 5.4%	PR 30%, SD 55% DCR 85% PFS 31.4mo, OS not reached at 38mo	Nicolini 2021 (29)
**PRRT+5-FU (200 mg/m2/24h)** [^111^In]In-Pentetreotide [^68^Ga]Ga-DOTATOC PET/CT [^18^F]F-FDG PET/CT	68	mNET	not reported	OS 72.1% and 52.1% at 2 and 5 years, respectively	Kong 2014 (30)
**PRRT + 5-FU (200mg/m2/24h)** [^111^In]In-Pentetreotide [^68^Ga]Ga-DOTATOC PET/CT [^18^F]F-FDG PET/CT	52	mNET	1xG4 thrombocytopenia 2xG3 thrombocytopenia 1xG3 liver failure	CR 2%, PR 28%, SD 68%, DCR 98% Metabolic response 27% Biochemical response 45% PFS 48mo, OS not reached at 36mo	Kashyap 2015 (31)
**PRRT+ CAP (1650 mg/m2/d/14d)** [^111^In]In-Pentetreotide	7	GEP-NET	1xG3 thrombocytopenia	Not Reported	van Essen 2008 (45)
**PRRT +TEM (150-250mg/m2) + CAPTEM (500-1000mg/m2)** [^68^Ga]Ga-DOTATOC PET/CT [^18^F]F-FDG PET/CT	2 12	mNET mNET	1xG4 liver failure 4xG3 liver failure	DCR (CT) 55% DCR (FDG) 38% DCR (Ga-DOTATOC) 44% PFS 7.1mo, OS 25.3mo	Yordanova 2019 (32)
**PRRT + 5-FU (200 mg/m2/24h)** **PRRT + CAP (1500mg/b.i.d.)** [^111^In]In-Pentetreotide	27 2	mNET	1xG4 lymphopenia 1xG4 late anaemia and thrombocytopenia	OS 34mo	Hubble 2010 (46)
**PRRT + CAP (1650 mg/m2/d/14d)** [^111^In]In-Pentetreotide	33	mNET	1xG3 thrombocytopenia 3xG3 angina	PR 24%, SD 70%, PD 6% DCR 94%	Claringbold 2011 (50)
**PRRT + CAPTEM** **CAP (1500 mg/m2/d/14d)** **TEM (100-200 mg/m2/d/5d)**	35	mNET	1xG3 nausea/vomiting 2xG3 neutropenia 2xG3 angina	CR 15%, PR 38%, SD 38% DCR 91% PFS 31mo, OS not reached at 24mo	Claringbold 2012 (51)
**PRRT+CAPTEM** **CAP (1500 mg/m2/d/14d)** **TEM (200 mg/m2/d/5d)**	30	pNET	3xG3 thrombocytopenia	CR 13%, PR 67%, SD 20%, Response rate 80% PFS 48mo, OS not reached at 33mo	Clairingbold 2016 (52)
**PRRT + CAPTEM** **CAP (1500 mg/m2/d)** **TEM (200 mg/m2/24h)** [^111^In]In-Pentetreotide	12/56	mNET (unknown primary)	1xG3 HFS	PFS 10.8mo in Grade 2 PFS 7.0mo in Grade 3	Chauhan 2018 (54)

Abbreviations: PRRT, peptide receptor radionuclide therapy; CAP, capecitabine; TEM, temozolomide; mNET, metastatic neuroendocrine tumour; MTC; medullary thyroid cancer; Pheo/Para, pheochromocytoma/paraganglioma; pNET, pancreatic neuroendocrine tumour; CR, complete remission; PR, partial remission; SD, stable disease; PD, progressive disease; DCR, disease control rate; PFS, progression-free survival; OS, overall survival; GEP, gastroenteropancreatic; HFS, hand-foot syndrome; PET, positron emission tomography; CT, computed tomography; FDG, fluorodeoxyglucose.

**Table 1B:** Combination of PRRT with CHEMOTHERAPY - Ongoing Prospective Studies (Status 1.9.2023)

Treatment Combination	Centre/Sponsor	n	Patients	Study Phase	No trial (reference)	Status
**PRRT+ CAPTEM vs CAPTEM alone** **PRRT+ CAPTEM vs PRRT alone**	Australia	72	pNET mid gut mNET	2	NCT02358356 (56)	Completed
**PRRT + Capecitabine vs PRRT alone (FDG-positive GEP-NET)**	Italy	35	GEP-NET	2	NCT02736448 (57)	Unknown
**PRRT+CAPTEM**	Poland	25	mGEP-NET	2	NCT04194125 (58)	Unknown
**PRRT + Capecitabine**	Italy	37	mGEP-NET	1/2	NCT02736500 (59)	Unknown
**PRRT+Capecitabine**	Sweden	300	mNET	3	NCT05387603 (60)	Not yet recruiting
**PRRT+Carboplatin, Etoposide, Tislelizumab**	Novartis	39	ES-SCLC	1	NCT05142696 (61)	Recruiting

Abbreviations: PRRT, peptide receptor radionuclide therapy; CAP, capecitabine; TEM, temozolomide; pNET, pancreatic neuroendocrine tumour; mNET, metastatic neuroendocrine tumour; mGEP-NET, metastatic gastroenteropancreatic neuroendocrine tumour; FDG, fluorodeoxyglucose; ES-SCLC, extensive stage small cell lung cancer

**Table 2:** Combination of PRRT with PARP Inhibitors - Ongoing Prospective Studies (Status 1.9.2023)

Treatment Combination	Centre/Sponsor	n	Patients	Study Phase	No trial (reference)	Status
**^177^Lu-DOTATATE + Olaparib** **p.o. 2 days before to 4 weeks after PRRT**	NIH, USA Bethesda, Maryland	37	GEP-NET	1/2	NCT04086485 (72)	Recruiting
**^177^Lu-DOTATATE + Olaparib** **dose escalation study (3 doses) 100 + 200 + 300 mg/d, 18 days**	Netherlands Erasmus Medical Center	24	locally advanced or mNET (G1-3)	1	NCT05870423 (73)	Recruiting
**177Lu-DOTA-TATE + Olaparib**	Gothenburg, Sweden	18	SSRT-positive tumours	1	NCT04375267 (74)	Unknown
**^177^Lu-DOTATATE+Talazoparib** **dose escalation study (4 doses) 0.1, 0.25, 0.5 and 1 mg/d/days 2-6**	Australia Peter MacCallum Centre	24	mNET	1	NCT05053854 (75)	Recruiting

Abbreviations: GEP-NET, gastroenteropancreatic neuroendocrine tumour; mNET, metastatic neuroendocrine tumour; SSRT, somatostatin receptor

**Table 3:** Combination of PRRT with Checkpoint Inhibitors - Ongoing Prospective Studies (Status 1.9.2023)

Treatment Combination	Centre/Sponsor	n	Patients	Study Phase	No trial (reference)	Status
**PRRT+ Nivolumab** **(240 mg iv, d1+d15/28d cycle)**	Spain (Multicentre)	30	NET G3 or NEC	2	NCT04525638 (81)	Active^*)^
**PRRT+ Avelumab** **(10 mg/kg/ 2 we/24 mo)**	Australia (Multicentre)	38	Merkel Cell Cancer	1/2	NCT04261855 (82)	Recruiting
**PRRT+ Pembrolizumab** **vs Pembrolizumab+TAE** **vs Pembrolizumab+RE**	University California, USA	32	mNET (Ki>20%, liver burden<75%)	2	NCT03457948 (83)	Active, not recruiting
**PRRT+ Pembrolizumab** **(400 mg/6 we/24 mo)**	Weill Medical College, Cornell, USA	18	Merkel Cell Cancer	2	NCT05583708 (84)	Recruiting

*) some data published. Abbreviations: NET, neuroendocrine tumour; NEC, neuroendocrine carcinoma; mNET, metastatic neuroendocrine tumour; TAE, transarterial embolization; RE, radio embolization.

**Table 4:** Combination of PRRT with Ribonucleotide Reductase, Tyrosin Kinase, DNA-dependent Protein Kinase or DNMT Inhibitors - Prospective Ongoing Studies (Status 1.9.2023)

Treatment Combination	Centre/Sponsor	n	Patients	Study Phase	No trial (reference)	Status
**^177^Lu-DOTATATE+ Triapine** **p.o. days 1-14**	NIH, USA	29	mNET	1	NCT04234568 (86)	Active, not recruiting
**^177^Lu-DOTATATE+ Triapine**	NCI, USA	94	NET	2	NCT05724108 (87)	Recruiting
**^177^Lu-DOTATATE + Peposertib p.o. days 1-21**	NIH, USA	29	pNET	1	NCT04750954 (89)	Recruiting
**^177^Lu-DOTATATE + Sunitinib malate p.o. days 1 - 28**	NIH, USA	24	pNET	1	NCT05687123 (90)	Recruiting
**^177^Lu-DOTATATE + Cabozantinib malate p.o. escalating 20, 40, 60 mg**	Oregon, USA	90	mNET	1	NCT05249114 (91)	Recruiting
**^177^Lu-DOTATATE+ ASTX727 Cedazuridine 100 mg + Decitabine 35 mg days 0-5**	London, UK	27	NET	1	NCT05178693 (92)	Recruiting

Abbreviations: mNET, metastatic neuroendocrine tumour; pNET, pancreatic neuroendocrine tumour; NET, neuroendocrine tumour

**Table 5:** Improvement of PRRT by Different Concepts

Concept	Centre/Sponsor	n	Patients	Study Phase	No trial (reference)	Status
**PRRT + ^131^I-MIBG**	Iowa, USA	50	GEP-NET	1/2	NCT04614766 (99)	Recruiting*)
**Neoadjuvant PRRT (2cycles) + surgery + PRRT (2cycles)**	Standford, USA	10	mGEP-NET	1	NCT04609592 (103)	Recruiting
**Neoadjuvant PRRT + surgery**	Milano, Italy	31	pNET	2	NCT04385992 (104)	Completed
**New SSTR-based radioligand** ^177^Lu-OPS201	IPSEN	40	mNET	1/2	NCT02592707 (116)	Terminated
**combination with other radioligands** **^177^Lu-DOTATOC+^161^Tb-DOTA-LM3** 0.5-1 GBq + 0.5-1 GBq „cross-over“design	Basel, Switzerland	16	mGEP-NET	1	NCT05359146 (122)	Recruiting
**New SSTR-based radioligand** ^212^Pb-DOTAMTATE	Radiomedix	33	mNET	1	NCT03466216 (125)	Unknown
**Combination of i.v. and i.a. routes (2 cycles i.a. then 2 cycles i.v.)**	Memorial SKCC, USA	10	mGEP-NET, bronchial or unknown NET	1	NCT04544098 (131)	Recruiting
**Combination of i.v. and i.a. routes (i.a. PRRT after 4 cycles i.v. PRRT)**	Bordeaux, France	20	mGEPNET	2	NCT04837885 (132)	Recruiting

*) some data published. Abbreviations: PRRT, peptide receptor radionuclide therapy; mGEP-NET, metastatic gastroenteropancreatic neuroendocrine tumour; pNET, pancreatic neuroendocrine tumour; mNET, metastatic neuroendocrine tumour
